# What are the lessons from COVID-19 for creating healthy, sustainable, resilient future cities?

**DOI:** 10.1038/s42949-023-00107-y

**Published:** 2023-06-02

**Authors:** Billie Giles-Corti, Sarah Foster, Bella Lynch, Melanie Lowe

**Affiliations:** Healthy Liveable Cities Lab, Centre for Urban Research, RMIT University, Melbourne, VIC Australia

**Keywords:** Environmental studies, Sustainability

## Abstract

The COVID-19 pandemic has disrupted lives and the economy, reminding the global community of the devastating health and economic impacts of uncontrolled infectious disease. It has affected how and where people live, work, shop, and play, and exposed our cities’ vulnerabilities, leading to calls for a health lens to be applied in designing, approving, and evaluating city plans. Socioeconomic, spatial and health inequities have been amplified, particularly for those living in inadequate or poorly designed housing, neighbourhoods, and cities. Hence, city mayors have committed to ‘build back better’ with all daily living amenities within a 15-min walking or cycling trip. Designed well, these cities have the potential to be healthier, more sustainable, equitable, and resilient. Yet their delivery requires a rethink of city planning. Drawing on lessons from the COVID-19 pandemic, we argue that to reduce the risk of future pandemics, we must mitigate climate change, limit urban expansion, and use nature-based solutions to protect natural habitats and biodiversity. We then explore how healthy, sustainable, and resilient 15-minute cities could be planned to reduce emissions and ensure our cities are more resilient in the event of future crises. Given that higher density housing underpins the success of 15-minute cities, we also examine how to create more resilient housing stock, through well-implemented health-supportive apartment design standards. Finally, we argue that to achieve all this, cross-sector leadership and investment will be vital.

## Introduction

The foundations of contemporary town planning, civil engineering and public health were built on the leadership of social reformers in the late 19^th^ century, advocating comprehensive interventions to curb morbidity and mortality associated with people living in overcrowded, unsanitary, and polluted industrialising cities^1–3^. Integrated infrastructure and policy interventions provided access to clean water and sanitation and improved housing conditions, the latter by requiring minimum lot sizes and separating noxious land uses from residential areas^4^. Prompted by 20^th^ century infectious disease epidemics, further improvements to higher density housing were achieved through regulations promoting natural light, ventilation and space^5,6^. Hence, in many cities – particularly those in higher income countries with well-implemented environmental health programs – infectious, waterborne and respiratory diseases have appeared to be largely tamed, and the associated morbidity and mortality prevented or controlled.

The COVID-19 pandemic has challenged this assumption^7^. Over three years into the pandemic, there have been over 600 million confirmed cases of COVID-19, and over 6.4 million deaths globally^8^. Irrespective of a city’s location or wealth, COVID-19 has disrupted lives and the economy, reminding the global community of the devastating human health and economic impacts of uncontrolled infectious disease^7^. Socioeconomic inequities have been amplified, particularly for those living in inadequate or poorly designed housing, neighbourhoods and cities^6,9^. The pandemic has affected how and where people live, work, shop and play, and exposed our cities’ vulnerabilities, with calls for a health lens to be applied to planning, approving and evaluating city plans^6,10,11^. Moreover, as human health is underpinned by eco-system health, COVID-19 has highlighted the need for the integrated, unifying and balanced approach to promoting health known as ‘One-Health’, that aims to optimise the health of people, animals *and* the environment^12^.

Given current and future global challenges confronting urban populations, planning cities to mitigate and adapt to future pandemics, climate change, and disasters must be a priority. The impacts of the pandemic have highlighted the need for cities to be resilient and designed to support urban dwellers to withstand future shocks^9^. Consistent with the World Health Organization’s concept of ‘One-Health’^12^, the UN Habitat^13^ defines a resilient city as one that ‘assesses, plans and acts to prepare for and respond to hazards - natural and human-made, sudden and slow-onset, expected or unexpected’ in ways that can protect and enhance people’s lives. This paper explores what lessons can be applied from this 21^st^ century pandemic, to create healthy, sustainable, resilient cities in the future?^6^

**Fig. 1:**
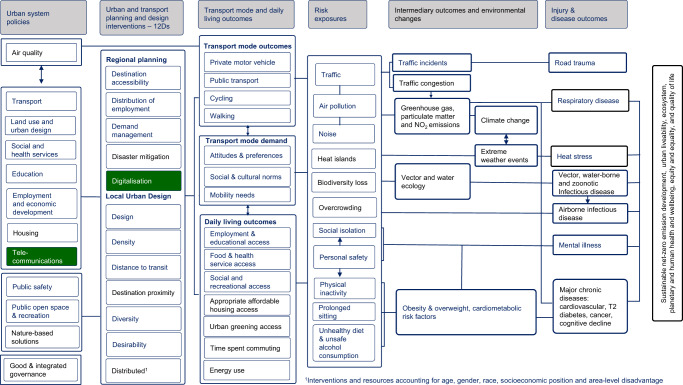
Pathways through which city planning policies and interventions affect health and can be used to optimise the 15-minute city. Modified from^14^ with new pathway indicated in green.

To address this question, we draw on a slightly modified framework of the pathways through which city planning impacts health, recently published in The Lancet Global Health series on urban design, transport and health (see Fig. 1)^14^. This comprehensive framework was developed in response to the significant interrelated challenges confronting cities in the 21^st^ century including population growth, rapid urbanisation, traffic congestion, rising non-communicable disease rates, transport-related air and noise pollution, climate change and biodiversity loss^14,15^. It takes a systems approach to city planning^16^ and considers the integrated upstream policies and interventions needed to optimise compact urban development that fosters human, ecosystem and planetary health. Compact urban development intensifies the use of space within a city through higher density housing in walkable neighbourhoods with mixed land use, public open space and efficient multi-modal transport infrastructure^17^. Done well, compact urban development fosters healthy and sustainable lifestyles. Done poorly, it can expose residents to individual, social and environmental risks, and impedes human, eco-system, and planetary health outcomes.

This framework is relevant to both non-communicable and communicable disease risks^14^ and could usefully guide policies to create resilient cities that protect residents’ health in the event of future crises. Rather than focussing on one sector, it addresses the upstream multi-sector ‘causes of the causes’ of poor health and environmental outcomes and proposes the need for integrated cross-sector planning and interventions. Hence, this paper applies and expands the framework to highlight the role city planning could and should play in promoting healthy and sustainable lifestyles in the post-COVID-19 era, by mitigating future crises (e.g., preventing future novel infectious diseases)^11,18^, creating built environments that support citizens to endure periods of lockdown and/or quarantine, and minimising the social, economic and health inequities that COVID-19 made so apparent.

This paper begins by exploring how the COVID-19 pandemic exposed the vulnerabilities of our cities for health and wellbeing; before considering the city planning features and lessons that could be applied to ensure future cities are designed to protect the health of *all* citizens in the event of a pandemic or other future crises.

## The impact of the COVID-19 pandemic on city dwellers

COVID-19 spread quickly throughout urban areas, particularly amongst the more vulnerable, and those living in crowded and poorly ventilated housing^7,14,19,20^. Without vaccines to protect citizens from serious illness, hospitalisation and death, governments worldwide introduced basic public health and social measures (PHSM) (e.g., handwashing, mask wearing, physical distancing and quarantine). In many cases, mandatory city-wide lockdowns restricted movement outside the home to essential activities such as food shopping, outdoor exercise and recreation, medical care and caregiving, and study or work that could not be done at home. Those classified as ‘essential’ workers, often disproportionately residing in disadvantaged neighbourhoods^21^, remained mobile across the city, increasing their risk of infection and transmission within and outside the home, particularly for those living in crowded households^19^.

Densely populated cities increase social interactions in public places and hence, potential exposure to infectious diseases. Initially, population density was implicated in accelerating disease transmission^10^. However, subsequent studies have challenged this assumption, observing that once strict PHSM were enforced, irrespective of the level of density, it was overcrowded living conditions^22^ and intra-urban mobility^23^, particularly by public transport^10^, that affected disease transmission and hospitalisation, rather than density-levels *per se*^24,25^. Indeed, some of the densest cities globally successfully curbed transmission by strictly enforcing PHSM^10,26,27^.

Nonetheless, as lockdowns were extended, the challenges of living, working, and studying in poorly designed housing, with insufficient or inflexible space, or in neighbourhoods that lacked key local infrastructure, such as green spaces or shops, increased social isolation and affected mental health^18^. This prompted an exodus to outer suburban and regional areas^26^, placing pressure on housing affordability and local infrastructure, and displacing lower income households, particularly in regional cities^28^. If unchecked post-pandemic, this trend could exacerbate the urban sprawl already rife across many cities^26^, and widen socioeconomic inequities^28^.

Working from home was enabled by digital technology that saw telecommuting for office workers become normalised. With most people working and studying from home, commuting and private motor vehicle use plummeted, and cities worldwide saw rapid declines in traffic, greenhouse gas emissions and air and noise pollution^25^. This was fortuitous because studies subsequently found an increased risk of COVID-19 transmission for those exposed to air pollution^25,29^. Air pollution is already the 4^th^ leading cause of global mortality and morbidity^14^, causing almost 9 million premature deaths annually^30^.

When exercise and essential errands were the only permitted activities outside the home, access to local shops and services and public open space were vital, and there were calls for more road space to be allocated to commerce, active transport and recreation to enable physically distanced circulation in the public realm^31^. This prompted a proliferation of ‘pop-up’ bicycle lanes in cities worldwide, providing opportunities for safe physically distanced travel^32^, particularly when people were fearful of disease transmission when using public transport. Such was the importance of cycling as a safe, effective and reliable form of recreation and transportation, cities such as Copenhagen classified bicycle shops as ‘essential services’^31,32^. The preference for walking and cycling during lockdowns highlighted ‘that non-motorised transportation systems are more resilient during pandemics’^25^, and must be prioritised post-pandemic, to create more resilient cities. Indeed, with more people working from home and spending more time in their neighbourhoods, urban dwellers and decision-makers alike began to appreciate the importance of living locally, with local access to shops, services, public open spaces, and multi-modal transport systems.

## Lessons for city planning

So, what lessons can we take from this experience, to protect urban dwellers during future pandemics or other crises? Here we reflect on and apply the Fig. 1 framework for creating healthy, sustainable, and resilient cities post-pandemic. First, we consider how city planning could mitigate future pandemics by reducing the impacts of urban development on natural habitats and avoiding biodiversity loss and climate change. Second, we explore how creating 15-minute cities that enable local living and sustainable mobility would reduce transport-related emissions and mitigate climate change while also enabling urban dwellers to adapt when confronted with a crisis, such as a pandemic. Finally, given higher density housing underpins achieving 15-minute cities, we consider how to ensure that higher-density housing is health-supportive, particularly in the event of a crisis.

## Protecting habitats and reducing biodiversity loss

Urban expansion is threatening biodiversity by ‘polluting, degrading and fragmenting habitat and displacing endemic species with introduced ones’ (p e923)^14^. The pandemic has been a timely reminder of the interconnection between humans and nature, given COVID-19 is a probable zoonotic disease that transferred to humans from an animal source^33^. Established socioecological models of health situate ecosystems and nature as fundamental determinants of healthy urban populations^2,34^. Emerging infectious diseases are a growing global health concern^35^, driven by biodiversity loss, which increases interaction and disease ‘spill over’ between species^33,36^. Once a new disease has appeared, crowded urban areas are perfect environments for transmission. Conversely, intact ecosystems reduce the risk of pathogen emergence and transmission among humans and animals^35,36^.

To prevent future epidemics and pandemics, policies that foster *nature-based solutions* are needed (see Fig. 1). This includes curbing urban expansion to reduce *biodiversity loss* and human activity encroaching into wildlife habitats, and biodiversity-sensitive urban design. Economic activity and resource use in cities also needs to limit environmental alterations and biodiversity loss well beyond urban boundaries, from pollution, deforestation, agriculture, resource mining and climate change^14,33,37^.

Climate change exacerbates the risk of infectious disease emergence and spread (including zoonotic, vector-borne, and water-borne disease) due to ecosystem disruption and the increased frequency and intensity of extreme weather and disasters (e.g., heatwaves, flood, bushfires)^33,35,38^. Exposure to multiple disasters, such as a heatwave or flood during a pandemic, has compounding impacts on health and inequities^20,39,40^. Transitioning to zero-emission cities is therefore imperative for disaster mitigation, and preventing the health impacts of climate change.

The COVID-19 pandemic brought a short-term dividend of reduced air pollution and emissions in cities, but these climate benefits need to be sustained and accelerated in the longer-term^20^. Cities also need to minimise impacts of climate-change related disasters, especially for vulnerable populations who are less able to adapt and respond. Key components of disaster risk minimisation are development controls in areas prone to disasters such as fire or flood, and provision of resilient infrastructure and housing that can withstand extreme weather^14,38^.

Policies that foster nature-based solutions and promote urban greening to integrate and protect nature within urban areas, not only make cities and neighbourhoods more desirable, but also have co-benefits for biodiversity, wildlife corridor protection, climate change adaptation, and resilience to urban heat, which impacts human health^14,25,41^. Well-designed urban green spaces played a vital role in urban resilience during the COVID-19 pandemic, enabling socially distanced outdoor recreation and contact with nature, with multiple population health benefits^25,42^. Thus, the size and equity of access to biodiverse green spaces and tree canopy cover should be key city planning considerations, along with biodiversity-sensitive design principles^14^. Planning resilient 15-minute cities post-pandemic is an opportunity to embrace the close connection between planetary and human health, and adopt an integrative approach to economic recovery and urban policy that safeguards biodiversity and ecosystems^33^.

## Enabling local living through 15-minute cities

Healthy and sustainable cities are underpinned by compact urban development that enables citizens to undertake daily activities locally using active forms of transport^14^. Yet for decades, low-density car-centric planning has dominated the design of cities worldwide, robbing residents of the health and other benefits afforded by active transport access to local amenities^43^. As the impacts of the pandemic became apparent, Moreno et al.^32^ and others^6,9,10,25,43–45^, have argued for a rethink of city planning to reduce inequities and ensure that urban dwellers’ basic needs - working, commerce, healthcare, education and entertainment - can be met locally by walking, cycling or micro-mobility. Indeed, with more people now working from home enabled by digital tools and infrastructure, the importance of local neighbourhoods for fulfilling daily needs has re-emerged. For example, C40, the global network of city Mayors, has committed to ‘build back better’ by creating 15-minute cities that support local living and prioritise active, sustainable mobility with co-benefits for reducing urban inequities, improving public health, and climate change mitigation^46^. Creating ‘cities of villages’ could make cities more resilient to future pandemics, enabling residents to adapt and thrive during lockdowns and potentially reducing geographic spread of disease associated with mobility^22^.

However, transitioning to 15-minute cities will require a new typology in the way cities are structured^25,32^, focussing on decentralised^47^, ‘proximity-based’ planning where all basic services required for daily living are available within 15 min by walking or cycling^9,44^. Moreno et al.^32^ have argued that achieving the 15-minute city requires a focus on four Ds: Density (i.e., ensuring sufficient population to make shops, services and public transport viable, but as discussed below, this must be done well to protect the health and wellbeing of residents), Destination Proximity (i.e., creating a city of short-distances where shops and services are within a walkable catchment), Diversity (i.e., of housing to achieve increased population density and social diversity, and diversity of destinations to make local living achievable) and Digitalisation (i.e., access to high-quality digital infrastructure that enables more people to work from home at least on some days of the week^48^).

All these Ds are vital. In addition to Moreno et al.’s 4Ds (three were already in our framework; and we added their Digitalisation), we include four regional intervention Ds (Destination accessibility (i.e., enhancing public transport to regional employment and activity centres), Distribution of employment (i.e., creating poly-centric cities with diverse employment opportunities that reduce commute distances, enable sustainable mobility, and increase the potential to work locally on at least some days of the week), Demand management (i.e., reducing the convenience and increasing the cost of driving and parking); and Disaster mitigation (i.e., restricting urban development in flood and fire prone areas; and using nature-based solutions that mitigate flood risk and protect natural habitats and biodiversity)) (Fig. 1). We also include four additional urban design Ds: Design (i.e., movement network design for a multi-modal transportation system that prioritises infrastructure and space for active transport^31^); Distance to transit (i.e., to facilitate public transport use); Desirability (i.e., urban greening that ensures sufficient access to public open space, while protecting biodiversity and habitats and increasing tree canopy that contributes to urban cooling); and Distributed (i.e., that ensures equity of access to all of the other Ds).

However, the success of compact urban developments that deliver the 15-minute city will be determined by the quality and resilience of its high-density housing stock. Given pressures on apartment dwellers during the pandemic, the next section focuses on lessons for optimising higher density housing.

## High-quality higher-density housing

Consistent with our framework, higher residential *Density is* essential to the creation of 15-minute cities as they provide the population needed to increase Destination proximity and decreases the Distance to a frequent public transport service^49^. However, the success and health impacts of the compact 15-minute city – particularly in a pandemic – also depend on the Design and Desirability of its high-density housing, and the level of Digitisation that allows residents to easily work from home^50^. This was exacerbated during the COVID-19 pandemic, with lockdowns and physical and social distancing restrictions increasing the ‘dose’ of exposure to the home environment, and accentuating the negative impacts of poor quality housing.

Like other housing types, poor apartment design and quality can expose residents to temperature extremes, inadequate ventilation, too little (or too much) sunlight, poor acoustic and visual privacy, and insufficient and/or inflexible space, with consequences for health and wellbeing^50,51^. However some of these design problems are heightened for apartment dwellers who typically have less control over indoor environmental conditions, less private indoor and outdoor space, and less flexible layouts^52,53^. This placed additional stresses on apartment dwellers during COVID-19, as there was limited capacity to socially distance within households to reduce disease spread, repurpose the space for home-based schooling, work or exercise (despite Digitalisation enabling home schooling and/or work), or retrofit apartments to address design problems that limited light, ventilation, thermal comfort or contact with nature^18,52–55^. Indeed, the pandemic re-emphasised the need for apartment standards that promote health and wellbeing, and building approval processes that ensure these requirements are implemented as intended^56^.

The nature of apartment living also affects social distancing *between* households within the building or apartment complex. Individual apartments are accessed via shared circulation spaces – lifts/elevators, stairwells, and corridors – that increase the potential for transmission between households, via contact with surfaces (e.g., door handles, lift call buttons) or closer physical proximity^52^. Communal spaces, such as outdoor gardens, provide additional space and respite, assuming they include the design features that make these spaces attractive and encourage their use (e.g., trees, greenery, seating)^57^. However, during COVID-19 peaks, many shared spaces were closed to residents to minimise infection^52^, confining residents to their apartment or forcing them into the wider neighbourhood, when permitted. While the closure of communal areas had worthy intentions, it had the potential to further penalise apartment residents, particularly those in smaller apartments where lockdowns increased mental distress^18^. Building designs that minimise physical proximity between residents (e.g., wider corridors, inviting staircases)^19,47^, improve natural ventilation (e.g., openable windows in internal circulation corridors)^56^, and incentivise larger communal areas with greenery would help minimise the impacts of future pandemics on apartment residents.

These policy settings for apartments and buildings are also important to adapt to extreme heat events associated with climate change (Disaster mitigation) and are vital for lower-income populations to reduce energy use and decrease the costs of mechanical heating and cooling^58^. However, healthy apartment design policy requires the support of wider neighbourhood planning policies that deliver nature-based solutions and preserve green space. These increase the Desirability of local neighbourhoods and the potential for green views, promote urban cooling that reduces heat islands^19,52^, and limit exposure to traffic *and* noise pollution^14^, all of which are important risk factors for health outcomes downstream (Fig. 1).

## Discussion

Transitioning to healthy and sustainable 15-minute cities is challenging. Yet cities and organisations around the world, including organisations such as C40, are committing to the concept^46^. As Nieuwenhuijsen^43^ points out, numerous urban models are already being implemented to improve established areas in cities through motorised traffic management and supporting active transportation in residential areas to reduce air and noise pollution and greenhouse gas emissions. This includes the Barcelona superblocks, London’s low-traffic neighbourhoods and Hamburg’s car-free city planning^43^. None of this is easy, but with courageous leadership, change is clearly possible. Creating resilient 15-minute cities for all will require a rethink of urban planning^32^, a commitment to delivering health-supportive high density housing, greater emphasis on the timely-delivery and financing of digital, and social infrastructure, and a compact city structure that ensures that all citizens have access to basic amenities required for daily living by active transport^25^. This necessitates a shift away from car-centric planning – whether electric, autonomous or not – and towards city planning that prioritises sustainable mobility: walking, cycling, public transport use and micro-mobility^59^.

Addressing complex city planning problems requires sector and academic silos to be broken down, and for ‘nexus’ or cross-sector integrated planning to be prioritised^14,15,60^. In nexus planning, each component is assessed without prioritising one over the other, to identify trade-offs and synergies to reduce the risk of negative externalities to another sector, duplication of efforts and resources^25^. Nexus planning requires good and integrated governance and shared budgets across government: hence, political will and leadership is vital to create an authorising environment that enables action and integrated planning across all urban system policies^15^.

Indeed, political will from all levels of government and the private sector is essential^61^. To achieve healthy, sustainable and resilient 15-minute cities for all requires investment in both green and social infrastructure. In the 21^st^ century, neighbourhoods are rarely built without clean water and sanitation. Similarly, green and social infrastructure must be elevated to ‘essential infrastructure’;^61,62^ and regulations and standards must ensure that human, eco-system, and planetary health are primary considerations in building new, and retrofitting existing, neighbourhoods and housing. Indeed, achieving healthy and sustainable 15-minute cities for all will require new legislation, regulations, and standards; cross-sector integrated horizontal planning across government departments, and vertical planning between different levels of government and the private sector as well as participatory planning with the community (particularly in established areas); and new co-funding arrangements across all levels of government to fund land and physical, digital, and social infrastructure development. Finally, inter-disciplinary research co-designed with policymakers and practitioners will be vital to optimise the 15-minute city, to benchmark and monitor implementation and to avoid any unintended consequences.
